# Supplementary material for: Sexual and gender identities and alcohol use during the COVID-19 pandemic
Source: PLoS One. 2024 Sep 6;19(9):e0308925. doi: 10.1371/journal.pone.0308925 (PMC11379183; doi:10.1371/journal.pone.0308925)
Supplement: Attachment — Submitted filename: Response to Reviewers2.docx [file pone.0308925.s002.docx]

**Journal Requirements:**

1. Please review your reference list to ensure that it is complete and correct.

We have corrected all errors in formatting and have checked to ensure that in-text citations match the reference list. We do not include any retracted articles.

**Additional Editor Requirements:**

1. Please review and make spelling checks throughout manuscript.

We have corrected any errors

2. Line 187, spelling correction for COVID-19

Corrected

3. Line 198 add “years” to Age ranged from 20 to 60

Added

4. Line 200 were surveys completed in Spanish included in the analysis?

We now clarify this in the paper: “The analytic sample of 3,593 includes English and Spanish	speakers…”

5. Line 242 check paragraph indentation

Done

6. Line 387 there is an extra period. cisgender men. .

Fixed
